# Caregiver perspectives on the impact of uncertainty on the everyday lives of autistic children and their families

**DOI:** 10.1177/13623613211033757

**Published:** 2021-07-22

**Authors:** Jane Goodwin, Priyanka Rob, Mark Freeston, Deborah Garland, Victoria Grahame, Ashleigh Kernohan, Marie Labus, Malcolm Osborne, Jeremy R Parr, Catharine Wright, Jacqui Rodgers

**Affiliations:** Newcastle University, UK

**Keywords:** anxiety, Autism Spectrum Disorder, intolerance of uncertainty

## Abstract

Approximately 50% of autistic children experience high anxiety. Intolerance of uncertainty (tendency to react negatively to uncertain situations) is a key mechanism in the development and maintenance of anxiety. This study aimed to provide a descriptive, thematic analysis of the impact on uncertainty on the daily lives of autistic children and their families. Semi-structured interviews were conducted with parents and caregivers of 53 autistic children (including 3 dyads) aged 6–16 years to elicit examples of uncertain situations that the children found challenging and to explore the impact of these situations on family life. Thematic analysis revealed five overarching themes: child’s reactions to uncertainty, trying to reduce uncertainty, the impact of difficulties with uncertainty, the impact of uncertainty on parenting and the impact on parents. These findings provide a crucial understanding into the range of uncertain situations autistic children with anxiety experience. Anxiety-reduction approaches based on increasing tolerance to everyday uncertain situations could help improve well-being, quality of life and participation for autistic children and their families.

**Lay abstract:**

Anxiety is common in autistic children. Research shows that this may be related to intolerance of uncertainty, which is a tendency to react negatively to uncertain situations. Understanding when, why and how autistic children respond to uncertainty is important in the development of anxiety programmes. We asked 53 (including 3 dyads) parents of autistic children about the types of uncertain situations that cause difficulties for their child and how uncertainty impacts on daily life for them and their families. We found that uncertain situations made autistic children and their families feel sad, worried, frustrated and angry through the themes: child’s reactions to uncertainty, trying to reduce uncertainty, the impact of difficulties with uncertainty, the impact of uncertainty on parenting and the impact on parents. There are lots of situations that are anxiety provoking for autistic children because of uncertainty, such as school. Programmes to reduce anxiety and increase autistic children’s ability to cope with everyday uncertain situations could improve quality of life for autistic children and their families.

## Background

Approximately 50% of autistic children experience high anxiety, consistent with an anxiety disorder (Simonoff et al., 2008; van Steensel et al., 2011), which significantly impacts on everyday life for them and their families. When anxiety is present in autistic people, anxiety is often complex, encompassing features of a range of anxiety disorders concurrently, alongside features not commonly found in non-autistic individuals (Kerns et al., 2014; Rodgers et al., 2017). A key mechanism in the development and maintenance of anxiety is intolerance of uncertainty (IU), which is a dispositional risk factor for the development and maintenance of anxiety (Carleton, 2012). IU can be a trauma response (Fetzner et al., 2013), which autistic people are more likely to experience (Rumball et al., 2020). Also, the environments autistic people are required to function in will contribute to and maintain IU: the double empathy problem (Milton, 2012) may increase uncertainty because autistic people are often required to modify their innate autistic social behaviours in order to adapt to, cope within and/or influence the predominately neurotypical social landscape (Cook et al., 2020). IU involves the ‘tendency to react negatively on an emotional, cognitive, and behavioural level to uncertain situations and events’ (Buhr & Dugas, 2009, p. 216). Intervention studies with neurotypical individuals suggest that a reduction in IU is associated with reduction in anxiety and improvements in everyday functioning (e.g. Boswell et al., 2013; Mofrad et al., 2020). Furthermore, cognitive behavioural treatments, which emphasise treating the cognitive process rather than the cognitive content of anxiety, specifically by aiming to increase an individual’s tolerance to uncertainty, achieve sustainable change (Hebert & Dugas, 2019; Wilkinson et al., 2011).

Recent research suggests that IU is an important construct associated with anxiety in autism (Boulter et al., 2014; Jenkinson et al., 2020) and is associated with some of the core characteristics of autism (Joyce et al., 2017; Rodgers et al., 2012; South & Rodgers, 2017). Understanding the contexts, responses to and impact of IU is critical to the development of appropriate interventions for anxiety among autistic children. In order to achieve this, this study reports a descriptive, thematic analysis of parents’ perceptions of the types of uncertain situations that cause difficulties for their autistic children, their responses to those situations and how IU impacts on family life.

## Methods

### Participant identification and recruitment

Data were collected as part of a feasibility and acceptability randomised pilot trial of a parent group-based intervention aimed at increasing tolerance to uncertainty in autistic young people: Coping with Uncertainty in Everyday Situations (CUES ©) (Rodgers et al., 2019). Parents or caregivers were specifically interviewed about their child’s IU to provide insight on a range of issues such as family stressors and coping strategies. Autistic children were not interviewed as these data are from a larger study focusing on parents. Parents were eligible if their child had an Autism Spectrum Disorder (ASD) diagnosis, was aged 6–16 years and experienced anxiety that interfered with their life in some way (determined by clinician). Parents or caregivers of autistic children with moderate to profound intellectual disability were not included in the study. The research was approved by the North East - Tyne & Wear South Research Ethics Committee (18/NE/0106).

The UK National Health Service multidisciplinary autism diagnostic and mental health teams received 424 study packs to distribute. A total of 80 families expressed interest in taking part in the trial. Of these, 28 were excluded (1 declined to participate, 25 could not be contacted and 1 sent an expression of interest form after recruitment had closed) and 2 withdrew after consent because they were too busy. Fifty-one families completed baseline assessments and progressed to randomisation (one family withdrew following randomisation because they felt CUES © did not suit them). Therefore, data for this study are from 50 families. Clinicians identified children who met inclusion criteria, discussed the study with the family and provided them with study packs. Interested parents completed an expression of interest form. The research team contacted parents to obtain written informed consent from the parent and assent from the child. Baseline data were collected, including a semi-structured interview with parents about uncertain situations. This article will report the findings from this interview. The participant characteristics are outlined in Table 1.

**Table 1.** Participant characteristics, grouped by child’s age band to protect participants’ privacy.

Child age band Number of children	Carer type (interviewee)	Child sex	Child school type	Vineland Adaptive Behaviour Scales Adaptive Behaviour Composite^a^ M (SD)	Anxiety Scale for Children – Autism Spectrum Disorder–Parent version Total score^b^ M (SD)
6–7 years 7 (14%)	7 Mother (14%)	6 Male (12%) 1 Female (2%)	7 Mainstream (14%)	72.8 (6.7)	35.4 (9.1)
8–11 years 30 (60%)	26 Mother (52%) 1 Father (2%) 2 Mother and father (4%) 1 Grandmother (2%)	22 Male (44%) 8 Female (16%)	20 Mainstream (40%) 8 Specialist school (16%) 1 ARC (2%) 1 Not attending school (2%)	65.1 (9.8)	35.3 (11.2)
12–14 years 9 (18%)	8 Mother (16%) 1 Mother and father (2%)	5 Male (10%) 4 Female (8%)	4 Mainstream (8%) 3 Specialist school (6%) 2 ARC^c^ (4%)	67.3 (5.2)	34.9 (12.5)
15–16 years 4 (8%)	4 Mother (8%)	2 Male (4%) 2 Female (4%)	2 Mainstream (4%) 1 College (2%) 1 Attending 2 schools (2%)	73.2 (8.1)	35.6 (12.2)

M: mean; SD: standard deviation.

a Range is 20–140. A score <100 indicates adaptive functioning level is below children of the same age.

b Score ⩾20 indicates significant anxiety levels.

c An ARC is an additionally funded specialist provision based in a mainstream school.

### Data collection

Parents were asked to identify two uncertain situations that their child found difficult in a semi-structured interview: one that was necessary (e.g. school), and one that the child wanted to do (e.g. parties). Children were not interviewed as it was parents who would be participating in the CUE session and also due to the young age of most of the children. A topic guide was developed for the semi-structured interviews. Topics included (1) the type of uncertain situation; (2) the child’s reaction (including symptoms and intensity); (3) how the uncertain situation interferes with daily functioning and activities for the child; and (4) how the uncertain situation interferes with daily functions and activities for the family (see Supplementary Materials for full topic guide). The aim of the trial was to explore intolerance of uncertainty, at recruitment and consent. Therefore, the child’s anxiety was predominantly due to uncertainty in the opinion of the referring clinician and the child’s parent. This was explored in detail with the interviewee, although participants did not usually have difficulty generating examples of IU. The interviews were conducted with participants in their home. They were audio recorded and averaged approximately 40 min long (range of 20–80 min). Participants also completed the Vineland Adaptive Behaviour Scales III (Sparrow et al., 2016), the Anxiety Scale for Children – Autism Spectrum Disorder–Parent version (Rodgers et al., 2016) and the Anxiety Disorders Interview Schedule with Autism Specific Addendum (Kerns et al., in press).

### Analysis

The analysis was informed by thematic analysis (Braun et al., 2019), which is an open and exploratory design and analytic process. It prioritises researcher subjectivity and reflexivity (Finlay & Gough, 2008; Gough & Madill, 2012). First, J.G. listened and relistened to the audio recordings which were transcribed verbatim. The transcriptions were then coded by authors J.G. and J.R. An inductive approach was used by identifying meaning from the interviews rather than using pre-determined codes to review and analyse the data. The codes were then combined into categories using thematic analysis. The analysis consisted of six stages (Table 2). Although there were a few discrepancies, any minor disagreements were resolved through robust discussion. Saturation was reached after 25 interviews, but we analysed 50 interviews with participants as they all provided valuable insight. *NVivo 12* (QSR International Pty Ltd, 2018) was used to manage the data.

**Table 2.** Stages of thematic analysis.

Research stage	Description of the research stage
1. Familiarisation with the data	J.G. listened to the audio recordings and read and re-read the transcribed interviews
2. Generating initial codes	J.G. and J.R. developed initial codes through discussion and definition of labels after coding the first few interviews
3. Searching for themes	J.G. and J.R. compared preliminary codes, collated the codes into potential themes and gathered all data related to each theme
4. Reviewing themes	J.G. and .J.R. reviewed the themes against the data set
5. Defining and naming themes	The themes were developed, revised and agreed on by all authors
6. Producing the report	Supporting quotes were selected to add in the report

### Community involvement

Autistic people and their families were involved and informed of all stages of the research. The author team comprises researchers, scholars, clinicians, advocates and community leaders. The author team also have personal experience as autistic adults, parents and caregivers of autistic children, and family members and friends of autistic individuals. Therefore, autistic adults and parents of autistic children were involved in the design and management of the research as co-applicants and members of the advisory group. The advisory group met regularly for consultation regarding specific aspects of the study including study documentation and the format of the semi-structured interviews. The topic guide for the semi-structured interviews was developed in collaboration with autistic individuals and with parents of autistic children. This was then refined after discussion with the rest of the research team. The themes from the analysis were discussed and clarified with all authors.

### Reflexivity and trustworthiness

All authors are researchers in autism and anxiety, as well as having personal experience as autistic adults, parents and caregivers of autistic children, and family members and friends of autistic individuals. V.G., J.R.P. and C.W. work clinically with autistic individuals and their families. While this expertise may have informed the author team to aspects of the participants’ experience, this personal and professional insight was a great strength during the open exploration of the data. J.G. conducted the interviews with participants, and in an attempt to deconstruct perceived imbalances between the ‘researcher’ and ‘participant’, the participants’ rights were emphasised from the outset, and it was made clear that the aim of the interview was to learn about their lived experience. J.G. as the interviewer was conscious throughout of bracketing potential biases. Without this conscious effort, biases could manifest as poor and/or judgemental interviewing techniques. During analysis, J.G. and J.R. delved into the participants’ accounts which required confronting their own experiences and biases. Coding of the transcripts (along with potential biases and reactions to the data) were discussed and clarified during supervision between J.G. and J.R., as well as with the other authors as a means of quality control and rigour check. The benefit of inductive coding is that unexpected topics could be noted and explored. The recordings were continuously referred to, to ensure we were staying true to the data. The transparent audit trail in *NVivo* accounted for the systematic examination at each level of analysis.

## Findings

Table 1 shows the children’s adaptive functioning and anxiety as rated by their parents. Table 3 shows the number of children who met clinical criteria for the *Diagnostic and Statistical Manual of Mental Disorders* (5th ed.; DSM-5) anxiety disorders. The types of uncertain situations that autistic children found difficult as perceived by participants are outlined in Table 4. Five overarching themes were identified: child’s reactions to uncertainty, trying to reduce uncertainty, the impact of difficulties with uncertainty, the impact of uncertainty on parenting, and the impact on parents Table 5 presents a summary.

**Table 3.** DSM-5 classification of children’s anxiety diagnoses.

Child diagnosis	*N* (%)
DSM anxiety disorders	
Generalised anxiety disorder	41 (82)
Specific phobia	42 (84)
Social phobia	27 (54)
Separation anxiety disorder	13 (26)
Panic disorder	2 (4)
Agoraphobia	5 (10)
Other anxiety disorder	1 (2)
At least 1 DSM anxiety diagnosis	49 (98)
ADIS-ASA specified conditions^a^	
Change^b^	
Fear of change	25 (50)
Negative reaction to change	14 (28)
Idiosyncratic phobia	17 (34)
Other social fear	8 (16)
Special interest fear	9 (18)
At least one ASD-ASA diagnosis	37 (74)
Other specified conditions^c^	
Post-traumatic stress disorder	2 (4)
Obsessive compulsive disorder	0 (0)
At least one other specified diagnosis	2 (4)

ADIS-ASA: Anxiety Disorders Interview Schedule – Autism Spectrum Addendum; ASD: Autism Spectrum Disorder.

a As defined by Kerns et al. (in press).

b Fear of change refers to both anticipatory fears of and poor reactions to change, novelty or rules. Negative reactions to change are coded only if the child has strong reactions to change but does not worry or expressive anticipatory anxiety (Kerns et al., in press). Children cannot receive a diagnosis of both fear of change and reactions to change.

c As defined by DSM-5.

**Table 4.** Types of uncertain situations reported by parents (two situations per child).

Uncertain situations	Examples	*N*
School	School	19
	Homework	4
	Tests/assignments	3
Extra-curricular activities	After school clubs	9
	Hobbies	3
	Swimming	3
Shopping	Shopping	6
	Going into town (includes sensory uncertainty)	2
New situations	New places	6
	Spontaneous/surprise events	1
	Watching new TV/movies	1
Family days out	Family days out	6
	Restaurants	3
Staying out or having people over	Mum going out	4
	Staying out	3
	Spending time at dad’s	1
	Friends coming over	1
Social situations	Parties (includes sensory uncertainty)	4
	Playing with friends	1
Spending time in neighbourhood	Walking in neighbourhood	3
Appointments	Medical appointments	3
Public transport	Catching metro/bus	2
Sensory	Busy/noisy places	1
	Clothes	1
	New foods	1
Routine events	After school	1
	Bath time	1
	Being alone (e.g. going to toilet)	1
	Games with family	1
	Meals	1
	Going in garden – insects	1
	Mum cooking	1
	Routines	1
	Failure	1

**Table 5.** Summary of themes and subthemes.

Theme	Sub theme	Description
Child reactions to uncertainty	• Repetitive behaviour and questions • Distress	Children’s repetitive behaviours and questions increased when they were worried. They could also get upset or angry.
Trying to reduce uncertainty	• Avoidance • Taking control	Children tried to evade uncertain situations or make rules to reduce uncertainty.
The impact of difficulties with uncertainty on autistic children	• Self-image • Missing out on fun • Missing out on education • Friendships	Children felt bad about themselves. IU meant that they could not participate in activities they wanted to do or needed to do. Their worry affected their friendships.
The impact of uncertainty on parenting	• Adaptations and adjustments • Providing reassurance • Preparation • Avoidance	Parents did their best to accommodate their child’s needs around uncertainty, including avoiding uncertain situations, preparing for uncertain situations and providing reassurance.
Impact on parents	• Unable to relax • Tiredness • Sadness for child’s distress • Wanting a magic wand • Frustration • Fear for future	Parents felt as though they were constantly anticipating uncertain events, helping their child manage their reaction to uncertainty or providing reassurance following an uncertain event. This was exhausting and frustrating. They felt sad that their child found many situations so difficult and wished they could change the world to be more understanding of their child. Parents worried about the impact uncertainty would have on their child’s future.

IU: intolerance of uncertainty.

### Child reactions to uncertainty

#### Repetitive behaviours and questions

Parents described repetitive behaviours associated with the anxiety-provoking uncertain situations they identified. Often these were self-stimulatory behaviours (stimming) that increased as the child became more anxious, including rocking, jumping, running, hand or finger movements, chewing, or repeating idiosyncratic words/phrases such as ‘it’s not ready yet’. While the behaviours were exhibited regularly, parents reported increased frequency and intensity of the behaviours when the child was in an uncertain situation:

You can see when his anxiety comes out . . . He rocks . . . how that settee isn’t through the wall is beyond me, because he lifts it off the floor . . . (Mother of 12-year-old male)

Many parents reported repetitive questions also increased in frequency and intensity in response to uncertainty. These questions were usually about what to expect in the uncertain situation and whether something might change or go wrong. Parents reported that providing an answer (e.g. how long they would be at the supermarket) often did not reduce their child’s questions:

You’ve got to tell him how long we’re going for, where exactly we’re going, how long is he in the car, what’s he going to be doing . . . (Mother of 13-year-old male)

Parents reported that some children required specific responses or actions to their questions, and this increased in uncertain situations. For example, one child wanted his mother to jump every time she got out of the car and would be more insistent about this in uncertain settings.

#### Distress

Parents reported that distress related to uncertainty was sometimes so overwhelming for their child that they could ‘shutdown’. Children temporarily lost their ability to do or enjoy their usual activities such as concentrating, playing, socialising, speaking, walking, listening or processing what is happening around them. Sometimes the child’s distress showed itself in other ways, for example, shouting, crying, hitting, kicking, biting and stamping feet. This behaviour was directed towards themselves or others:

I think his ability to process information and interact, just virtually disappears when he’s anxious, so we try to minimise how much we talk to him and each other . . . (Mother and father of 9-year-old male)

Anger, everything comes out in anger. (She will) smash things up, yell, shout, scream, hit. She’s just like a whirlwind, anything that’s in her way is just smashed. (Mother of 9-year-old female)

### Trying to reduce uncertainty

#### Avoidance

Children did anything they could to avoid uncertain situations. For example, reporting illnesses (e.g. ‘I feel sick’), hiding (themselves or items required for the situation such as swimming gear), looking for alternatives (e.g. asking to be home schooled to avoid school), refusing to go to bed or get up in the morning and moving very slowly to delay the event. Some children tried more extreme measures such as trying to escape from moving cars. Parents frequently reported that if their child was unable to avoid the situation, they became worried, withdrawn, irritable, angry or distressed:

She just panics: you can see the fear in her face. (Mother of 11-year-old female)

Avoidance meant that children missed out on doing things they enjoy. Either they could not get there because the uncertainty was intolerable; or by the time they got there, they were so anxious (and worn out from their reactions to uncertainty) that they found it difficult to enjoy the activity:

She’ll want to spend more time on her tablet and she tries to refuse activities. She won’t want to go out, she won’t want to take part in family games or anything like that . . . She withdraws. (Mother of 10-year-old female)

#### Taking control

In order to reduce uncertainty, some parents reported their child created strategies to manage uncertain situations, such as using rules and conditions, usually around limiting time spent doing the activity or restricting choices for others (e.g. ‘which DVD out of these three will we watch?’). Children also gave themselves a particular role to reduce uncertainty (e.g. group leader), created their own rules (e.g. a game has to be played in a certain way) or directed themselves/others (e.g. ‘I have to be first in the pool’), or the physical environment:

He’ll give set instructions . . . five minutes this, five minutes that . . . he wants to be in control of the situation, and it’s like, we can’t keep on with the five minutes! (Mother and father of 10-year-old male)

It’s just controlling everything to help his worry. (Mother of 9-year-old male)

One child checked potential visitors on social media before deciding whether her mother could have friends over. This reliance on preparation or taking control could backfire and become extremely distressing if anything changed.

### The impact of difficulties with uncertainty on autistic children

#### Self-image

Many parents reported that their child had low self-esteem, often because difficulties with uncertain situations meant that children worried about many things. Some of the children saw that their peers did not worry about things in the same way they did. Each time an uncertain situation was too much to manage, children lost confidence and felt disappointed:

He can sometimes cry. He starts talking negatively about himself . . . about life. (Mother of 11-year-old male)

Children often felt guilty and embarrassed about their reactions to uncertainty, and worried about the impact on their family. Sometimes children also reported uncertainty in relation to their own abilities. For example, if a child was doing some homework, they may not believe that the notes they took during class were correct:

He sees some people get divorced because of the kids. So he worries that maybe (his worry about) school . . . is just making trouble between me and dad. (Mother of 13-year-old male)

He feels like he can’t trust what he’s written. He will do the bare minimum because he isn’t sure what to write, especially when the tasks are not black and white. (Mother of 11-year-old male)

#### Missing out on fun

Anticipation of an uncertain situation made it difficult for a child to enjoy their usual activities. One parent reported that uncertainty related to school meant that their child was only ever happy on Saturdays, because there was no school that day or the next. Other children had their leisure time interrupted by their uncertainty related worries:

He’ll stop what he’s doing and ask his questions. Sometimes he’ll just pause his telly and come down and ask them. (Mother and father of 8-year-old male)

Similarly, when children were in an uncertain situation, the children found it hard to ‘live in the moment’ because they were still so worried about the uncertainties. Sometimes uncertainty meant the child was too anxious to go to an event, or they had to leave early, which resulted in disappointment and sadness:

Once he’s there, he’s enjoying it; but if it’s a thing where you move from one thing to the next, like a theme park, it could be ‘that ride was good but I want to go on there’ and then he’d say it again and again . . . so it’s the anticipation of what comes next . . . and then ‘when are we going, when are we going?’ (Mother of 12-year-old male)

He will be very excited . . . then when it comes to the day of (he’ll say) ‘I really want to go, I really want to go’ and then he’ll get there (and he’ll say) ‘I want to go home, I want to go home’. (Mother of 6-year-old male)

#### Missing out on education

Difficulties with uncertainty also impacted on education. Parents reported their child found certain aspects of the school day to be too uncertain (e.g. which lessons will be on that day and how the child will perform in a test). Even when the child was able to physically join the class, they were often too anxious to be able to concentrate and process the information that they would need to be able to mentally access the course content, which would then stop them from completing their work because they were so unsure about their answers:

He must be missing out . . . I just want him to go back to school . . . two whole years without education is horrific. (Mother of 9-year-old male)

Everything stops at that point . . . she won’t do much of anything. She gets herself so wound up and so anxious about it. (Mother of 11-year-old female)

Children missed out on education beyond school as well. Many children wanted to attend extra-curricular activities, such as swimming and music. However, the uncertainty kept them from taking part:

He’s in total anguish. He doesn’t know why he’s feeling like he is . . . It’s got to stop him in some ways, because we have tried so many different things and we haven’t find anything that sticks yet. (Mother of 14-year-old male)

#### Friendships

Difficulties with uncertainty also impacted on opportunities to form friendships, with parents reporting that their child was often unable to participate in activities such as after school clubs and school trips due to the uncertainty of the situation. Some parents reported that established relationships had faltered because of their child’s difficulties with uncertainty:

I think she has missed out, because . . . the groups of friends she had . . . a lot of them went (on school trips), and of course they bonded more, and I think as a result she became a more of an outsider. (Mother and father of 13-year-old female)

It can affect his friendships . . . he’ll be shorter with his friends and may get more irritated by them, get upset and not be able to cope with friendship squabbles – they would escalate a lot quicker and he would isolate himself. (Mother of 11-year-old male)

### The impact of uncertainty on parenting

#### Adaptations and adjustments

Many parents accommodated their child’s anxiety by adjusting their family routines, engaging more than usual with schools (e.g. meetings and phone calls to find answers to questions), preparing, setting aside additional time for support, reassuring, debriefing and avoidance or non-attendance at events:

We involve him in the menu planning . . . and we would make those meals . . . We have had to completely and utterly rework everything we do . . . it’s quite a big undertaking (laughs) . . . We have to sit down and talk about all this (meal anxiety) on a regular basis to see where we’re at, what’s working, what leaks have sprung from other places. It’s like a constant check. (Father of 10-year-old male)

#### Providing reassurance

Parents reported that they needed to provide a lot of reassurance before, during and after uncertain events. During the event, parents encouraged and supported their child to keep going. Often the parents used distraction to help their child, such as topics of conversation that are interesting to the child, special toys and snacks. If the parents were not there, they usually identified a safe person for the child to go to, such as a trusted teacher:

I’ll distract him by talking or I might just mention something about a game that he’s after . . . then as soon as I talk about that he’s oblivious. So I know in my mind that I’m sort of like trying to be one step ahead of him all the time. (Mother of 15-year-old male)

Children also experienced uncertainty after events and ruminated on what happened. Parents often needed to talk through the event with their child and analyse various interactions and reassure their child that they would not get in trouble for what happened (e.g. accidentally bumping into someone), that it was fine to get things wrong (e.g. answers on tests) and that people still liked them:

Whether it’s reassuring or . . . go through every single technique in the book every day when he’s at his worst to try and like rationalise it to him. It does take an awful lot of time. Sometimes we get him to write down or draw out things. (Mother of 13-year-old male)

#### Preparation

Parents tried to prepare as much as possible for uncertain situations. The planning often started with providing as much advanced warning as possible. Preparation also included using visual maps, adding items to the child’s schedule board and researching:

We gave him plenty of warning . . . And we just sort of kept reminding him about how many days it was, then the day before or the morning of . . . (Mother of 10-year-old male)

The real extreme lengths we have to go to plan everything, and just the time and the energy and effort that takes. It’s a lot of work . . . Sometimes he’ll look at pictures . . . he’ll go on Google maps and see pictures of the roads and shops and things. (Mother and father of 9-year-old male)

To best prepare their child, parents often spent a lot of time doing their own research. Parents felt the need to be one step ahead, and they often went through all the details with their child, and re-explained the details many times in the lead up to the situation. Parents attempted to answer any questions their child had, and if they did not know the answers, they called the organisers (e.g. leaders of after school club) to find out all the details. Parents also worked hard to make sure that all the people their child spoke to would have a consistent message. Another aspect of preparation was practising being in the uncertain situation at home or in a safe space. Parents introduced their child to things that worried them; for example, they may cook menu items from a restaurant at home:

I talk her through . . . go through the whole scenario – where mummy’s going, when mummy’s coming back, mummy’s gone before and always come back, and all those scenarios. (Mother of 10-year-old female)

#### Avoidance

A lot of the time, parents reported that they would avoid uncertain situations that made their child anxious. For example, if the child was scared of new places, they would avoid new places ‘at all costs’:

There might be that one element that he can’t cope with very well, and you think it’s not worth taking a risk. (Grandmother of 11-year-old male)

### Impact on parents

#### Unable to relax

Parents reported they frequently struggled to find time to wind down or relax because they spent a lot of time preparing for and managing the child’s reaction to uncertainty. Even when the child was out at an event they were excited about, the parent felt on edge:

There’s times where I think that I’m going to be picking her up but actually she’s ended up having such a lovely time that . . . she might walk back in the group because she’s feeling quite comfortable and then I think oh I could’ve actually had a glass of wine. It’s just my evening revolving around the what-ifs and buts. (Mother of 16-year-old female)

#### Tiredness

Parents regularly felt exhausted. They had little respite from planning around the child’s uncertainty, being hyper vigilant during uncertain situations and having to manage the child’s reactions to uncertainty. Many parents were unable to leave their child with a trusted person (e.g. grandparents) due to the child’s worries. Parents spoke of having ‘occupied’ minds and always having to be ‘mindful’ of what might affect their children. This was draining and this tiredness impacted on other areas of life, such as relationships and motivation:

It’s very hard. (When they leave for school) I sit behind the door for 15 mins, 20 mins like this just to calm down . . . And then I start another worry of something happening when dad dropped him. (Mother of 13-year-old male)

I’ve lost me sparkle. It’s definitely had a huge impact on me. It’s the second time I’ve been on my happy pills . . . I’m still not quite in the frame of mind of doing things – lack of energy, exhausted, lack of motivation. Trying to pick the easy road. Constantly thinking. (Mother of 10-year-old female)

#### Sadness for child’s distress

It was extremely upsetting for parents to see their child experience such distress on a regular basis. They wished things could be easier for their child. It was especially upsetting when other children managed the same situations with ease. Many parents described seeing their child’s anxiety as ‘heart-breaking’. It was particularly sad when others did not accept their child. It was hard for parents to see their child struggle to overcome their anxiety (e.g. building up the courage to attend an event) and see them rejected socially:

I wish she was more easy. And it’s hard because I don’t think people see what a lovely girl she is and it doesn’t look like – she absolutely loves the horses, she loves the stables – so perhaps people don’t realise that. It’s hard to see people impatient or irritated. (Mother of 14-year-old female)

#### Wanting a magic wand

Parents wished that the world would be more tolerant and understanding of their child. It was frustrating and upsetting to see their child experience so much distress regularly. Many parents described the desire to wave a ‘magic wand’ to solve their child’s problems. They wanted to take their child’s worries away (‘fix it all’) and ‘make everything better’ or ‘easier’ for them. Some parents wanted to use their ‘magic wand’ to get support for their child. Other parents wanted to use the ‘magic wand’ on themselves to give them more time in the day to help their child, or have the parenting skills to stop the child experiencing anxiety, such as the ability to read their child’s mind:

We worry for her. We wish we could read what her mind is thinking, what’s ticking inside her head. I think we worry about – are we supporting her in the right way? (Mother and father of 10-year-old female)

#### Frustration

Parents also experienced irritation or frustration related to the child’s difficulties with uncertainty. Parents found it particularly difficult when there was an activity or event they knew their child could do and would enjoy, yet it caused the child a lot of worry. It turned something that should have been positive into a ‘hugely negative thing’. Furthermore, sometimes the repetitive questions caused frustration because parents could not always provide answers that would satisfy their child:

It’s just frustrating because you know that it’s stuff that he will enjoy but eventually and its stuff that’s good for him to be able to do. (Mother of 6-year-old male)

We tell him the same answer every week because he’s asking questions that we can’t answer. We don’t know what instructor you’re going to have, we don’t know if you’re going to get moved up a stage, we don’t know what pool you’re going to be in. And still he asks the questions every week. (Mother and father of 8-year-old male)

Parents also reported feeling frustrated at the support available and with themselves for not always having the solutions or tools available to help their child and change the environment:

Helpless . . . yes . . . sometimes a bit angry as well . . . with those who make his life hard . . . Sometimes angry as well with the services because . . . I find if the services were targeting the needs he has, he wouldn’t be having some of these problems. (Mother of 11-year-old male)

#### Fear for future

Parents worried about how their child’s difficulties with uncertainty would affect them in the long-term. Life is full of uncertainties that children will need to manage independently in the future, and parents did not always know the best way to support their child. They did not want their child to be limited in the activities they want to do or have to do. Also, parents were concerned that their child’s anxiety would increase with age as they would be exposed to more uncertain situations. For example, parents thought if their child worried this much about their weekly spelling quiz, how would they manage major exams?

He’s 16 and he should be able to go into town . . . I don’t think he can lead an independent life, if I’m honest. (Mother of 16-year-old male)

I just worry about her when she gets older, ‘cause like, when you’re teenagers and you get anxiety, it just hits you like a brick, really’. (Mother of 9-year-old female)

It was hard for parents to find the balance between encouraging their child to try new things and keeping them in their ‘comfort zone’. Some parents reported staying away from uncertainty increased their child’s difficulties:

I think he’s wasting his abilities . . . and the opportunity to enjoy life, enjoy things that he likes . . . and it’s reinforcing the rigidity and the limits his life already has, so it’s keeping himself within those limits instead of expanding and having wider experiences that he needs. (Mother of 11-year-old male)

## Discussion

This is the first study to explore the caregiver’s perception of the types of uncertain situations that caused difficulties for autistic children with anxiety and how these difficulties impacted on their daily lives and those of their families. Caregivers reported a variety of situations that covered the full range of school, social, routine care, extra-curricular activities, travelling, everyday tasks, special occasions, and outings and trips. Across the 100 uncertain situations identified by the 50 interviews, uncertainty impacted on all aspects of the lives of young people and their families. Parents spontaneously and most commonly identified school-related situations in exacerbating their child’s intolerance to uncertainty and increasing anxiety. Uncertain situations in school included changes in routine, different teachers, getting answers wrong, getting in trouble, what to do at break time and tests. Teachers also perceive increased anxiety in their autistic compared to non-autistic students (Syriopoulou-Delli et al., 2019) and parents report anxiety related to uncertainty for their autistic children in school contexts (Adams et al., 2019). Other uncertain situations identified included extra-curricular activities, shopping and new situations in general. The thematic analysis revealed five overarching themes related to autistic children’s reactions to uncertainty, ways they would try to reduce uncertainty and how it affected them.

Parents reported that restricted and repetitive behaviours such as repetitive questions, rocking, jumping, playing with hands (often known as self-stimulatory behaviours or stimming) and inflexibility increased when their child was experiencing anxiety related to uncertainty. Although these behaviours are a core feature of autism, previous research has indicated that heightened anxiety is associated with increased engagement in these behaviours; perhaps as a way to make the world more predictable or as a form of internal emotion regulation (Joyce et al., 2017; Manor-Binyamini & Schreiber-Divon, 2019 ; Rodgers et al., 2012). Indeed, a longitudinal study has demonstrated that children who showed behavioural rigidity (e.g. strict adherence to routines) at preschool age were more likely to experience both insistence on sameness and elevated anxiety at 8–11 years of age (Baribeau et al., 2021).

It is clear from our findings that difficulties with uncertainty caused autistic children and their families a lot of worry and distress. Parents reported that children often missed out on opportunities for enjoyment, friendship and education had low self-image and low mood. Furthermore, difficulties managing everyday uncertain situations that other children seemed to cope well with increased feelings of ‘being different’ and ‘not belonging’, which in turn may provide increased risk of further mental health difficulties (Pelton & Cassidy, 2017).

Difficulties with uncertainty also impacted on family life, with parents providing multiple examples of family accommodation to reduce their child’s anxieties, through reassurance, changes to routines and avoidance of specific people, places or activities (Lebowitz et al., 2013), supporting previous findings (Adams & Emerson, 2020; O’Connor et al., 2020). Understandably, parents wished to reduce the child’s distress, but they also told us how they themselves found uncertain situations tiring, frustrating and upsetting and that they would often take the ‘easiest’ path. These findings clearly demonstrate the importance of a family approach to addressing difficulties with uncertainty (Adams & Emerson, 2020). Recent developments of parent mediated interventions to address difficulties with uncertainty for autistic children show promise in addressing the impact of these difficulties on everyday life for both autistic children and their parents (Rodgers et al., 2017, 2019).

### Strengths and limitations

Study limitations include focusing on parent perspectives of the impact of uncertain situations rather than collecting first person accounts from the children themselves. Research by Joyce et al. (2017) shows that young people aged between 13 and 20 years were able to describe uncertainty and their own anxiety. Future studies should gather insights from young people themselves, and this could possibly strengthen or contradict our findings. We focused on caregiver perspectives because these data are from a larger study supporting parents to support their child. Future research should include fathers’ perspectives as well as other caregivers’ experiences. Furthermore, the inclusion criteria did not include families of autistic children with moderate to profound intellectual disability, and it is important for future research to determine the impact of uncertainty on these children and their families. Finally, this is a relatively large number of participants for thematic analysis; however, due to the breadth of the research question, the diversity of participants’ experiences and variability in the richness of data (Braun et al., 2019), this sample size was considered appropriate. Data were collected from a treatment-seeking sample as participants were families that were accessing the National Health Service (NHS) services for diagnosis and mental health support. While this means that the families have extensive experience of the impact of uncertainty on their everyday lives, the findings may not generalise to non-treatment seeking children.

## Conclusion

Our findings indicate that parents perceive a range of everyday uncertain situations challenging and anxiety provoking for their autistic children. These are situations that occur in typical daily life and can cause increased distress, worry, frustration and anger, lowered mood and self-image. Uncertainty may lead to missed opportunities for learning and fun, while also impacting on parental energy and well-being. Interventions to reduce anxiety and increase autistic children’s ability to cope with everyday uncertain situations could improve quality of life for autistic children and their families. Exploring the difficulties autistic children and young people experience with uncertainty may enhance our understanding of the link between intolerance of uncertainty and anxiety in autistic children and young people.

## Supplemental Material

sj-docx-1-aut-10.1177_13623613211033757 – Supplemental material for Caregiver perspectives on the impact of uncertainty on the everyday lives of autistic children and their families Click here for additional data file. Supplemental material, sj-docx-1-aut-10.1177_13623613211033757 for Caregiver perspectives on the impact of uncertainty on the everyday lives of autistic children and their families by Jane Goodwin, Priyanka Rob, Mark Freeston, Deborah Garland, Victoria Grahame, Ashleigh Kernohan, Marie Labus, Malcolm Osborne, Jeremy R Parr, Catharine Wright and Jacqui Rodgers in Autism
