# Supplementary material for: Caregiver perspectives on the impact of uncertainty on the everyday lives of autistic children and their families
Source: Autism. 2021 Jul 22;26(4):827–38. doi: 10.1177/13623613211033757 (PMC9014769; doi:10.1177/13623613211033757)
Supplement: sj-docx-1-aut-10.1177_13623613211033757 – Supplemental material for Caregiver perspectives on the impact of uncertainty on the everyday lives of autistic children and their families [file sj-docx-1-aut-10.1177_13623613211033757.docx]

# Addressing intolerance of uncertainty in children with autism spectrum disorder: An intervention feasibility trial

## *Target Situation Semi-Structured Interview*

## Child Impact

- 1. Is there a situation that your child would like to do on a regular basis that cause them (and you) the most stress or difficulty because of uncertainty (or unknown elements)?
  2. Is there another situation that causes your child (and you) the most stress or difficulty because of uncertainty (or unknown elements), but it is appropriate and necessary? (Although if they had a choice they wouldn’t do it)

1. What about the situation is uncertain?
2. How often does this occur?
3. How long has your child experienced IU about this situation?
4. How does your child react?
5. Could you describe the intensity and symptoms of this reaction?
6. How long does this reaction last?
7. Does your child worry about the situation before it happens? *(Prompt: How can you tell?)*
8. How does this IU interfere with daily functions and activities for your child?
   1. What extra things does your child need to do because of this IU?
   2. What can’t your child do because of this IU?
   3. What is the emotional impact of this IU?
   4. How has this IU impacted on relationships?

## Family Impact

1. What extra things do you need to do because of this IU?
2. What can’t you do because of this IU?
3. What is the emotional impact of this IU?
4. What is the impact of this IU on the child’s siblings?
5. How has this IU impacted on relationships?
